# Validation of the Surgical Outcome Risk Tool (SORT) and SORT v2 for Predicting Postoperative Mortality in Patients with Pancreatic Cancer Undergoing Surgery

**DOI:** 10.3390/jcm12062327

**Published:** 2023-03-16

**Authors:** Anna P. Karamolegkou, Maria P. Fergadi, Dimitrios E. Magouliotis, Athina A. Samara, Evangelos Tatsios, Andrew Xanthopoulos, Chryssa Pourzitaki, David Walker, Dimitris Zacharoulis

**Affiliations:** 1 Department of Anesthesiology, Hippocration General Hospital of Athens, 11527 Athens, Greece; 2 Department of Radiology, University of Thessaly, Biopolis, 38221 Larissa, Greece; 3 Division of Surgery and Interventional Science, Faculty of Medical Sciences, University College London, London WC1E 6AU, UK; 4 Department of Surgery, University of Thessaly, Biopolis, 41110 Larissa, Greece; 5 Department of Cardiology, University of Thessaly, 38221 Larissa, Greece; 6 Department of Clinical Pharmacology, Faculty of Medicine, School of Health Sciences, Aristotle University of Thessaloniki, 54124 Thessaloniki, Greece

**Keywords:** risk assessment, risk tool, sort, surgical outcome risk tool, pancreatic cancer

## Abstract

Background: Pancreatic cancer surgery is related to significant mortality, thus necessitating the accurate assessment of perioperative risk to enhance treatment decision making. A Surgical Outcome Risk Tool (SORT) and SORT v2 have been developed to provide enhanced risk stratification. Our aim was to validate the accuracy of SORT and SORT v2 in pancreatic cancer surgery. Method: Two hundred and twelve patients were included and underwent pancreatic surgery for cancer. The surgeries were performed by a single surgical team in a single tertiary hospital (2016–2022). We assessed a total of four risk models: SORT, SORT v2, POSSUM (Physiology and Operative Severity Score for the enumeration of Mortality and Morbidity), and P-POSSUM (Portsmouth-POSSUM). The accuracy of the model was evaluated using an observed-to-expected (O:E) ratio and the area under the curve (AUC). Results: The 30-day mortality rate was 3.3% (7 patients). Both SORT and SORT v2 demonstrated excellent discrimination traits (AUC: 0.98 and AUC: 0.98, respectively) and provided the best-performing calibration in the total analysis. However, both tools underestimated the 30-day mortality. Furthermore, both reported a high level of calibration and discrimination in the subgroup of patients undergoing pancreaticoduodenectomy, with previous ERCP, and CA19-9 ≥ 500 U/mL. Conclusions: SORT and SORT v2 are efficient risk-assessment tools that should be adopted in the perioperative pathway, shared decision-making (SDM) process, and counseling of patients with pancreatic cancer undergoing surgery.

## 1. Introduction

Pancreatic cancer (PC) represents a major cancer-related cause of death and is currently the fourth most common cause of cancer-related mortality in the USA [1,2]. Most of the cases diagnosed with PC are adenocarcinomas (PDAC) and are commonly located in the pancreatic head or neck [3,4]. In spite of the important advances in anticancer research, PC-associated mortality continues to rise and the prognosis continues to be poor. Thus, it is projected that by 2030, PC will represent the second-highest cancer-related cause of mortality [5,6], with most patients undergoing potentially curative surgery. The treatment strategy for pancreatic cancer should be multidisciplinary, including regimens of chemo- and radiotherapy in conjunction with surgery [7]. On this basis, there is an urgent need for an accurate assessment of the patient’s perioperative risk to facilitate shared decision-making (SDM) and the informed consent process while raising the standards of clinical practice quality on the perioperative pathway. In addition, the adoption of a specific and sensitive risk-stratification tool allows for the accurate comparative evaluation of surgical results among institutions, departments, and surgeons for either service evaluation or clinical audit. Several such tools have been implemented into clinical practice [8]. Despite the increasing interest in more advanced risk-stratification tools, risk prediction models remain the most easily accessible choice for this purpose. Nonetheless, they are not frequently employed in everyday practice, potentially due to poor awareness amongst clinicians and with concerns about their accuracy and complexity [9].

The Surgical Outcome Risk Tool (SORT) was proposed following the 2011 National Confidential Enquiry into Patient Outcome and Death (NCEPOD) report [9]. It was developed with the goal of providing a tool that could easily provide an enhanced level of risk stratification for surgical patients in a user-friendly manner [9]. In order to be user-friendly, SORT utilizes only six clinical data variables [9]. Currently, it has been compared favorably with other previously validated risk-stratification tools, such as the ASA physical status (ASA PS) grade, and has been externally validated in groups of patients undergoing hip fracture surgery [10] and colorectal surgery [11]. In both groups [10,11], SORT was associated with acceptable discrimination and calibration levels.

Our previous study implementing preliminary outcomes [12] was the first to validate SORT in patients undergoing surgery for pancreatic cancer, but we did not perform a comparison with other traditional risk-stratification tools. Furthermore, in that study [12], the number of included patients was limited. In addition, an updated version of SORT (SORT v2) has been developed that takes into consideration the physician’s risk estimation of the surgery [13]. In this context, the present study aimed to validate the SORT and SORT v2 models in adult patients undergoing surgery for pancreatic cancer and compare them with other traditional risk prediction models.

## 2. Methods

### 2.1. Data Extraction Strategy

The current study was performed according to a protocol designed and agreed upon by all authors. Data were extracted from a prospectively maintained database of consecutive patients with pancreatic cancer who underwent surgery between 1 January 2015, and 31 August 2022. All procedures were performed by a single surgical team led by the senior author (D.Z.) at the Department of Surgery, University Hospital of Larissa, Greece. Ethical approval was obtained by the Scientific Committee of the hospital (Protocol number: 50271/30-10-19). Informed consent was waived based on the retrospective nature of the present study. No imputation methods were employed for missing data.

We extracted and included data regarding age, gender, body mass index (BMI), ASA (American Society of Anesthesiology) grade, history of previous operations, operative priority, surgical severity, malignancy status, staging, and type of procedure. We defined mortality as any patient death that occurred during the first 30 days or during the hospital stay if longer than 30 days. The predicted risk of mortality was determined using the SORT and SORT v2 models. Moreover, the predicted mortality was calculated by employing POSSUM and P-POSSUM for all patients. In all cases where the patients’ data were incomplete, they were excluded from the analysis.

In order to identify the accuracy of each model, we performed separate sensitivity analyses. These additional analyses were performed to evaluate the discrimination and calibration traits of each model relevant to predicting the perioperative mortality risk based on (1) a procedure-related variable: surgical operation (pancreaticoduodenectomy or total pancreatectomy or distal pancreatectomy); (2) cancer-related variables: CA19-9 levels (≥500 mU/L vs. <500 mU/L), neoadjuvant treatment (received or not); and (3) patient-related variables: age (≥70 vs. <70), pre-operative ERCP (yes or no), and postoperative pancreatic fistula (POPF) (yes or no). The risk for POPF was assessed using the formula described by Weng et al. [14]. We employed these variables given that they might affect postoperative mortality.

### 2.2. Primary and Secondary Endpoints

The validation of the SORT and SORT v2 models in adult patients with PC undergoing surgery was set as the primary endpoint of the present study. Secondary endpoints included (1) the comparison of SORT and SORT v2 with the POSSUM and P-POSSUM models regarding their discrimination and calibration traits in predicting perioperative mortality and (2) a subgroup sensitivity analysis.

### 2.3. Statistical Analysis

The SORT score was calculated using the method and web platform developed and proposed by Protopappa et al. [9], in addition with the updated version incorporating subjective information to calculate the SORT v2 score [13]. The SORT and SORT v2 models implement five variables: ASA physical status, operative priority level (elective, urgent, immediate), surgical specialties (gastrointestinal, thoracic, or vascular surgery), surgical severity (major/complex), and malignancy status, age (65–79 or ≥80 years). Surgical severity is calculated automatically upon the entry of procedure details. According to the developers’ guidelines, if the procedure performed is not listed, the nearest available procedure is used for calculation [13]. The procedures from the list we used were “total pancreatectomy” and “distal pancreatectomy”, both associated with major severity. SORT v2 also implements the physician’s perceived mortality risk [13]. The POSSUM and P-POSSUM scores were calculated by employing the method proposed by Copland [15] and Prytherch [16], respectively.

Discrimination (the ability to distinguish patients who died from patients who did not die) and calibration (the ability to successfully predict the mortality rate) traits of the SORT and SORT v2 models were assessed. Discrimination was assessed by producing receiver-operating characteristic (ROC) curves and calculating the area under the ROC curve (AUC). The AUC was determined by calculating the 95% confidence intervals and was compared by employing nonparametric paired tests, as described by DeLong [17]. The model discrimination was defined as poor, fair, or excellent when the AUC was of <0.70, 0.70–0.79, and 0.80–1.00, respectively [17].

The calibration was calculated for each included model by measuring the expected mortality and then comparing it with the observed mortality. An observed-to-expected ratio of 1 represented perfect accuracy, a ratio < 1 represented an overestimation of mortality rate, and a ratio of >1 demonstrated an underprediction. Furthermore, calibration was also assessed by employing the Hosmer–Lemeshow (H-L) goodness of fit test, with a lack of fit defined as a *p*-value ≤ 0.05 [18]. In cases where the outcome variable separated the predictor variable completely, a perfect separation was described.

All extracted data were tabulated using Microsoft^®^ Excel 16.61 (Microsoft, Redmond, WA, USA) and were analyzed by employing Prism^®^ Graphpad 9.3.1 for Mac (GraphPad Software, San Diego, CA, USA).

## 3. Results

### 3.1. Baseline Patient Characteristics

**Figure 1:**
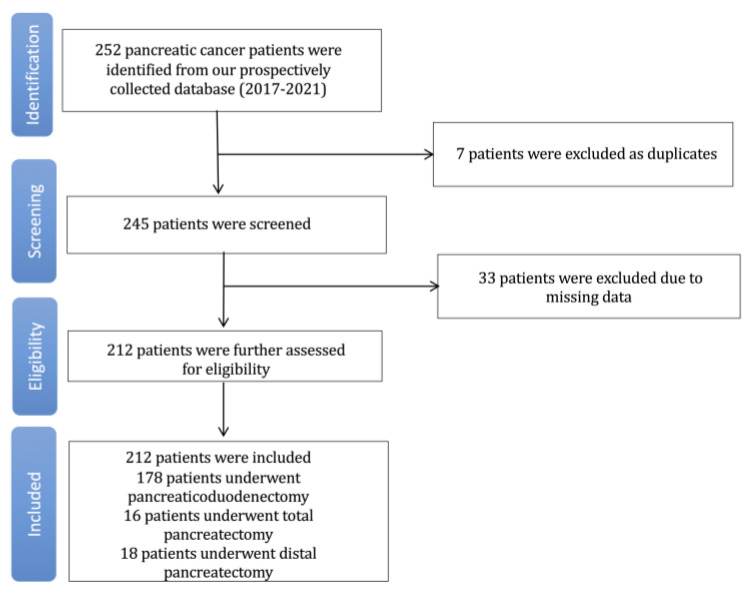
Trial flow.

The findings of the current study are presented in accordance with the STROBE (Strengthening the Reporting of Observational Studies in Epidemiology) guidelines [19]. The trial flowchart for the study, which demonstrates the data extraction strategy, is reported in Figure 1. In total, 252 patients were screened, and 212 patients were finally incorporated. The patients’ baseline characteristics are presented in Table 1. Of the total group, 78 (36.8%) female patients were included, with a mean age of 67.2 (standard deviation (SD)—10.5) years. Most of the cases presented with a re-sectable tumor (71.7%) and underwent an elective procedure (91.5%). The tumor was located primarily in the head (180 patients—84.9%) of patients. Most of the cases were PDAC 190 (89.6%), with a mean CA19-9 of 502.9 (SD: 1136) U/mL. A total of 178 (84%) patients underwent pancreaticoduodenectomy, sixteen (7.5%) a total pancreatectomy, and eighteen (8.5%) a distal pancreatectomy. Finally, the overall 30-day mortality rate was 3.3%.

### 3.2. Performance of SORT and SORT v2 Models in the Total Dataset

**Figure 2:**
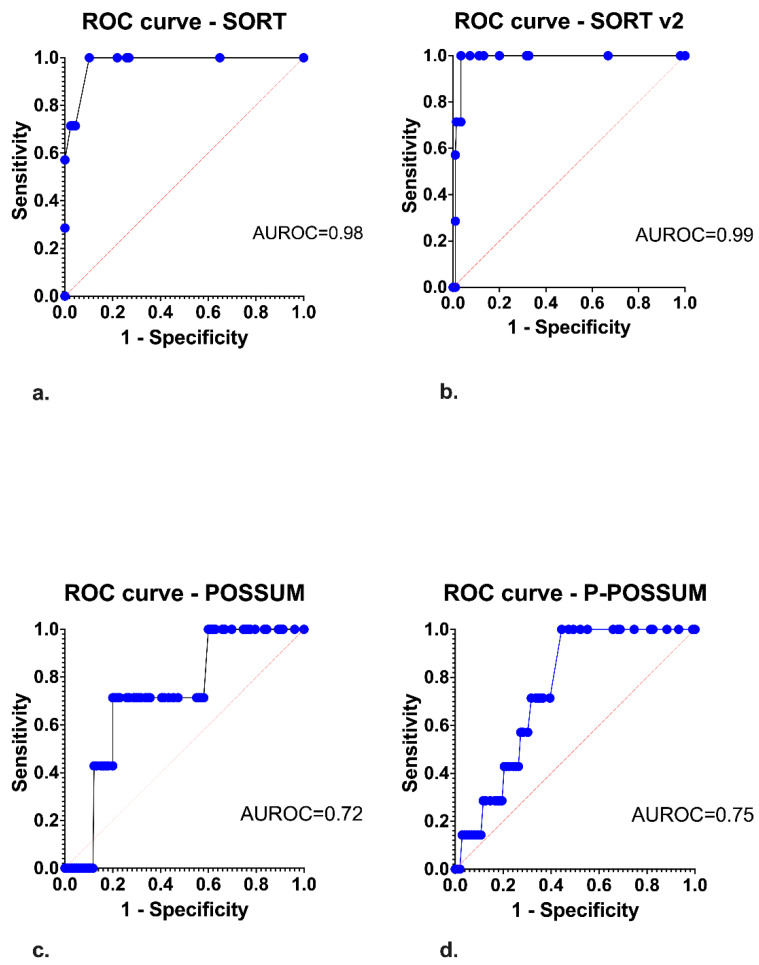
Receiver Operating Characteristic (ROC) curves regarding the discrimination of each model in the total study population. ROC curves regarding. (**a**). Surgical Outcome Risk Tool (SORT), (**b**). SORT v2, (**c**). Physiological and Operative Severity Score (POSSUM), (**d**). Portsmouth-POSSUM (P-POSSUM).

The performance of SORT is presented in Table 2 and Figure 2. In fact, SORT was associated with an excellent discrimination level in the total analysis (AUC: 0.98 (95% CI: 0.95–1.00); *p* = 0.001). SORT v2 presented similar discrimination (AUC: 0.98 (95% CI: 0.97–1.00); *p* = 0.001). Furthermore, SORT demonstrated the lowest Hosmer–Lemeshow value (H-L: 2.97; *p* = 0.71), thus showing the best-performing calibration for all models in the total analysis. SORT v2 demonstrated the second-lowest H–L value (H-L: 5.46; *p* = 0.49). Nonetheless, both SORT and SORT v2 underestimated the mortality determined by observed/expected ratios of >1.

### 3.3. Comparison of SORT and SORT v2 with Other Mortality Prediction Models in the Entire Dataset

The POSSUM (AUC: 0.72 (95% CI: 0.57–0.88); *p* = 0.045) and P-POSSUM (AUC: 0.75 (95% CI: 0.64–0.86); *p* = 0.025) were associated with a fair discrimination level (Table 2), though both underestimated mortality (Table 2).

### 3.4. Performance of Mortality Prediction Models in Subgroups

**Figure 3:**
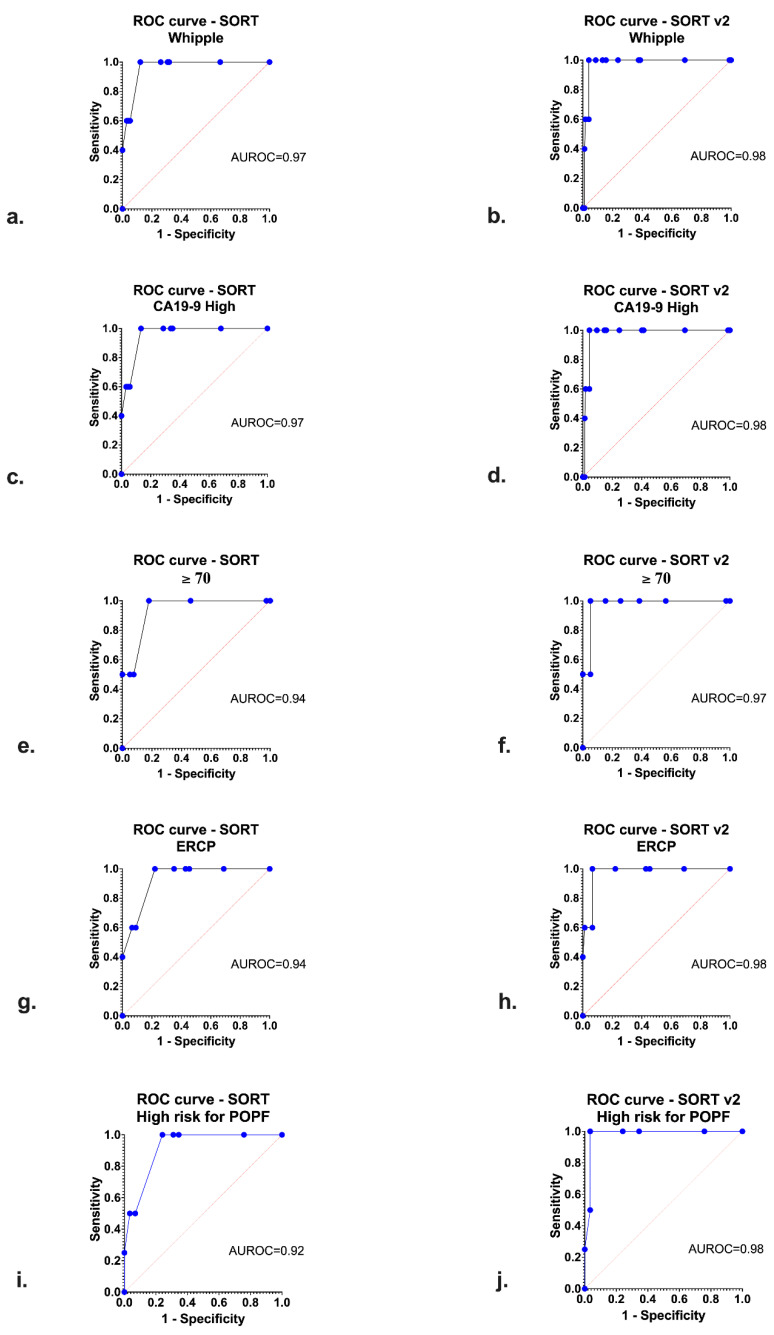
Receiver Operating Characteristic (ROC) curves regarding the discrimination of Surgical Outcome Risk Tool (SORT) and SORT v2 in the following subgroups: (**a**,**b**): pancreaticoduodenectomy procedure; (**c**,**d**): CA19-9 ≥ 500; (**e**,**f**): age ≥ 70; (**g**,**h**): pre-operative endoscopic retrograde cholangiopancreatography (ERCP); (**i**,**j**): High risk for postoperative pancreatic fistula (POPF).

The outcomes derived from the subgroup analysis are shown in Table 3 and Figure 3. The SORT and SORT v2 models demonstrated an excellent discrimination level in predicting perioperative mortality in all subgroups. In certain subgroups, SORT and SORT v2 models demonstrated a perfect separation, which is translated into a perfect prediction of mortality (Table 3). Furthermore, POSSUM and P-POSSUM were inferior in terms of the discrimination level in most of the subgroups when compared with SORT and SORT v2. In addition, SORT demonstrated a high level of calibration in all subgroups, with the lowest value reported in patients undergoing pancreaticoduodenectomy with high levels of CA19-9 and a previous ERCP. In all subgroup analyses except “ERCP or No ERCP”, SORT and SORT v2 underestimated the perioperative mortality.

## 4. Discussion

The current original trial represents the first attempt to validate SORT and SORT v2 models in (1) PC surgery and (2) compare them with additional traditional risk models such as POSSUM and P-POSSUM, and (3) perform a sensitivity subgroup analysis. This study also represents the first external validation of SORT v2 currently provided in the literature and especially in PC surgical patients. The outcomes provided by the present study directly affect daily clinical practice, suggesting the potential value of SORT and SORT v2 in the perioperative pathway and during the counseling and shared decision-making (SDM) processes for patients with PC scheduled for surgery.

SORT remains a useful and probably the most user-friendly risk-stratification tool. It was developed by Protopapa et al. [9], who aimed to accurately predict the 30-day mortality in an objective manner. The present trial demonstrated that six pre-operatively available clinical variables could efficiently predict postoperative mortality with a higher accuracy compared to other traditional risk assessment tools, such as ASA-PS [9]. In the same context, SORT v2 was proposed as an enhanced version of the original SORT as it implements the physician’s perception of the perioperative mortality risk [13]. Other risk-stratification tools that have been implemented in clinical practice and were included for comparison in the current study are POSSUM and P-POSSUM. Given that both patients and physicians have implemented these tools in the SDM process, it was important to compare them with SORT and SORT v2. In addition, according to recent evidence [15], traditional risk-stratification tools, such as POSSUM and P-POSSUM, were associated with poor accuracy, while new models are required to provide enhanced calibration and discrimination traits, according to findings derived from prospectively collected data [15]. Our outcomes provide a response to this call for enhanced risk-stratification models in the setting of PC surgery. SORT and SORT v2 demonstrated the best-performing discrimination and calibration characteristics compared with all other risk-stratification models assessed in the present study. Our outcomes not only follow the preliminary outcomes of our previous study [12] but also highlight the superiority of both tools compared with POSSUM and P-POSSUM and validate SORT v2 for the first time. In this context, the outcomes of this study have direct implications for the SDM process of patients with PC regarding their postoperative mortality risk, thus helping patients to co-shape their treatment strategy.

The efficiency of both SORT and SORT v2 was also demonstrated in the sensitivity subgroup analyses. SORT and SORT v2 were associated with excellent discrimination traits and enhanced calibration. However, we should further stress our comparative outcomes regarding patients undergoing pancreaticoduodenectomy with raised levels of CA19-9 and pre-operative ERCP. In this group, SORT and SORT v2 demonstrated excellent calibration and discrimination traits and showed significantly lower H–L values compared to POSSUM and P-POSSUM. Patients with these baseline characteristics represent the most difficult cases faced by our HPB multi-disciplinary teams. These are commonly symptomatic patients, diagnosed through a thorough diagnostic workup after presenting with jaundice. At that stage, they commonly present CA19-9 levels over 500 U/mL, thus demonstrating an aggressive tumor biology, although the tumor is borderline resectable in most of these cases. They also commonly undergo ERCP stenting to alleviate jaundice prior to surgery, especially in cases in which neoadjuvant treatment is chosen. In this context, it is of great importance to have access to such an effective and reliable risk-stratification tool during the MDT meetings when such complex cases are discussed, in addition to during the patients’ counseling process.

We have not found a significant difference between SORT and SORT v2, thus proposing that the physicians’ estimation of perioperative mortality risk does not significantly affect the original SORT outcomes. Nonetheless, in all analyses, SORT v2 demonstrated slightly better discrimination and calibration compared with SORT. Consequently, it would be interesting to investigate whether there is a discrepancy between SORT and SORT v2 in patients undergoing pancreaticoduodenectomy with vascular reconstruction. Despite our original intention to perform such a subgroup analysis, there were limited available cases that underwent pancreaticoduodenectomy with major vascular reconstruction to perform further analyses. Consequently, this clinically relevant question requires further investigation by a future trial mainly focusing on complex cases. Moreover, the findings of the current study regarding the value of clinical variables employed by SORT remain in accordance with the evidence provided by administrative datasets [20]. Finally, according to our outcomes, SORT and SORT v2 are associated with higher accuracy compared with other pre-operative (BH 2009—Barwon Health 2009) [21] and intraoperative risk-stratification tools (SAS—Surgical Apgar Score) [22], while remaining user-friendly as they implement six clinical variables.

Although POSSUM and P-POSSUM have been extensively validated [2], SORT and SORT v2 have certain advantages. To begin, both tools incorporate only six pre-operative variables, significantly fewer compared with the eighteen perioperative variables of POSSUM and P-POSSUM. They are thus significantly easier to implement in real-life clinical practice. Moreover, POSSUM and P-POSSUM include intra- and postoperative variables that are not available during the pre-operative assessment. Finally, (P-)POSSUM contains certain subjective variables, thus increasing the interobserver variability and heterogeneity and posing a certain bias.

The current study is associated with certain limitations. One limitation is associated with the study design, given that it is a single-institution retrospective trial. Nonetheless, it should be noted that all data was prospectively collected, the patients were consecutive, the surgical team remained the same, and the surgeon’s bias regarding patient or surgical approach selection was minimized as this was decided based on MDT suggestions and patients’ choices after extensive counseling. In addition, given that one of the most important postoperative complications associated with high morbidity and mortality in pancreatic surgery is POPF, there is a certain limitation related to the lack of this variable in the formulas of all the risk-stratification tools implemented in the present study.

The current outcomes demonstrate that SORT and SORT v2 are feasible, friendly, and efficient risk-stratification tools that should be implemented in the pre-operative counseling and SDM process of patients with PC undergoing surgery, thus enhancing clinical quality in a cost-effective manner. In addition, they are useful instruments to be taken into consideration during multidisciplinary meetings when examining complex cases associated with comorbidities and frailty.

## 5. Conclusions

In the present study, we validated the SORT and SORT v2 risk-stratification models in adult patients undergoing surgery for pancreatic cancer. Both tools demonstrated the best-performing discrimination and calibration compared with POSSUM and P-POSSUM. The value of SORT and SORT v2 was further confirmed by sensitivity subgroup analyses. Both tools are associated with excellent discrimination and calibration, especially in patients with PC undergoing pancreaticoduodenectomy with pre-operative ERCP and CA19-9 levels over 500 U/mL. SORT represents a feasible and efficient risk stratification tool that can be easily implemented in the perioperative pathway of patients with PC.

## Figures and Tables

**Table 1:** Patient baseline characteristics.

Baseline Characteristics	Number of Patients, n = 212
Female, n (%)	78 (36.8)
Mean age, years (SD)	67.2 (10.5)
Age ≥ 70 (%)	82 (38.7)
BMI, (SD)	26.5 (1.9)
Mean previous Operations, n (SD)	1.9 (1)
Pre-operative ERCP, n (%)	82 (38.7)
ASA Class, n (%)	
I	48 (22.6)
II	112 (52.8)
III	42 (19.8)
IV	10 (4.7)
Stage, n (%)	
Re-sectable	152 (71.7)
Borderline re-sectable	60 (28.3)
Mean CA19-9, U/mL (SD)	502.9 (1138)
CA19-9 ≥ 500 U/mL, n (%)	162 (76.4)
CA19-9 < 500 U/mL, n (%)	50 (23.6)
Neoadjuvant treatment, n (%)	56 (26.4)
Operative priority	
Elective	194 (91.5)
Acute	18 (8.5)
Cancer site, n (%)	
Head/Vater	180 (84.9)
Body	14 (6.6)
Tail	18 (8.5)
Pathology, n (%)	
PDAC	190 (89.6)
NET	14 (6.6)
Other	8 (3.8)
Surgical Operation, n (%)	
Pancreaticoduodenectomy	178 (84)
Total pancreatectomy	16 (7.5)
Distal pancreatectomy	18 (8.5)
30-day mortality	7 (3.3)

Abbreviations: ASA: American Society of Anesthesiologists; BMI: Body Mass Index; PDAC: Pancreatic ductal adenocarcinoma; NET: Neuroendocrine Tumor; CA19-9: Carbohydrate antigen 19-9.

**Table 2:** Discrimination and calibration traits for each score regarding the prediction of mortality in patients with pancreatic cancer undergoing surgery.

Scoring Systems	O	E	O:E	Discrimination		Calibration	
				AUC (95% CI)	*p*	H-L	*p*
POSSUM	7	0	-	0.72 (0.57–0.88)	0.045	17.47	0.03
P-POSSUM	7	0	-	0.75 (0.64–0.86)	0.025	9.47	0.31
SORT	7	4	1.75	0.98 (0.95–1.00)	<0.001	2.97	0.71
SORT v2	7	4	1.75	0.98 (0.97–1.00)	<0.001	5.46	0.49

Abbreviations: O: observed; E: expected; AUC: area under curve; 95% CI: 95% confidence interval; H–L: Hosmer–Lemeshow.

**Table 3:** Discrimination and calibration traits of each score for predicting mortality in certain subgroups.

Scoring Systems	O	E	O:E	Discrimination		Calibration	
				AUC (95% CI)	*p*	H-L	*p*
Pancreaticoduodenectomy (n = 178)							
POSSUM	5	0	-	0.67 (0.50–0.84)	0.193	9.56	0.297
P-POSSUM	5	0	-	0.75 (0.64–0.86)	0.055	11.18	0.192
SORT	5	2	2.5	0.96 (0.92–1.00)	<0.001	2.89	0.822
SORT v2	5	2	2.5	0.98 (0.95–1.00)	<0.001	6.87	0.443
Total pancreatectomy (n = 16)							
POSSUM	perfect seperation						
P-POSSUM	ps						
SORT	ps						
SORT v2	ps						
Distal pancreatectomy (n = 18)							
POSSUM	2	2	1	0.88 (0.71–1.00)	0.092	6.82	0.556
P-POSSUM	2	0	-	0.75 (0.54–0.96)	0.261	17.09	0.017
SORT	ps						
SORT v2	ps						
CA19-9 ≥ 500 mU/L (n = 162)							
POSSUM	5	0	-	0.69 (0.51–0.86)	0.157	9.30	0.318
P-POSSUM	5	0	-	0.76 (0.65–0.87)	0.048	9.88	0.274
SORT	5	2	2.5	0.96 (0.91–1.00)	<0.001	2.82	0.831
SORT v2	5	2	2.5	0.97 (0.95–1.00)	<0.001	6.76	0.562
CA19-9 < 500 mU/L (n = 50)							
POSSUM	2	0	-	0.79 (0.68–0.91)	0.166	18.94	0.015
P-POSSUM	2	0	-	0.71 (0.58–0.84)	0.322	15.29	0.054
SORT	ps						
SORT v2	ps						
Neoadjuvant treatment (n = 56)							
POSSUM	3	2	1.5	0.98 (0.95–1.00)	0.005	0.41	>0.999
P-POSSUM	3	1	3	0.95 (0.88–1.00)	0.009	21.89	0.003
SORT	3	2	1.5	0.91 (0.81–1.00)	0.018	0.83	0.997
SORT v2	3	2	1.5	0.98 (0.95–1.00)	0.005	3.63	0.822
No neoadjuvant treatment (n = 156)							
POSSUM	4	0	-	0.63 (0.42–0.85)	0.370	16.73	0.033
P-POSSUM	4	0	-	0.74 (0.62–0.86)	0.104	12.14	0.145
SORT	ps						
SORT v2	4	2	2	0.99 (0.9671.00)	<0.001	1.29	0.972
≥70 (n = 82)							
POSSUM	2	0	-	0.53 (0.26–0.79)	0.904	8.65	0.373
P-POSSUM	4	0	-	0.65 (0.45–0.84)	0.322	8.52	0.384
SORT	4	2	2	0.94 (0.86–1.00)	0.003	4.50	0.480
SORT v2	4	2	2	0.97 (0.94–1.00)	0.001	0.71	0.994
<70 (n = 130)							
POSSUM	3	0	-	0.92 (0.87–0.97)	0.014	22.27	0.004
P-POSSUM	3	0	-	0.82 (0.75–0.89)	0.059	12.24	0.141
SORT	ps						
SORT v2	3	2	1.5	0.98 (0.96–1.00)	0.004	1.18	0.947
ERCP (n = 82)							
POSSUM	5	4	1.25	0.83 (0.66–1.00)	0.001	16.71	0.033
P-POSSUM	5	2	2.5	0.89 (0.89–1.00)	0.001	6.88	0.550
SORT	5	2	2.5	0.93 (0.85–1.00)	0.001	2.23	0.973
SORT v2	3	2	1.5	0.97 (0.94–1.00)	<0.001	36.27	<0.001
No ERCP (n = 130)							
POSSUM	2	0	-	0.81 (0.75–0.88)	0.130	11.38	0.181
P-POSSUM	2	0	-	0.79 (0.63–0.95)	0.156	7.76	0.458
SORT	ps						
SORT v2	2	2	1	0.98 (0.96–1.00)	0.019	0.52	0.991
High risk for POPF							
POSSUM	4	4	1	0.94 (0.86–1.00)	0.005	6.22	0.622
P-POSSUM	4	2	2	0.91 (0.80–1.00)	0.009	5.64	0.688
SORT	4	3	1.33	0.92 (0.81–1.00)	0.008	0.88	0.997
SORT v2	4	3	1.33	0.98 (0.93–1.00)	0.002	17.81	0.013
Low risk for POPF							
POSSUM	3	0	-	0.59 (0.34–0.84)	0.59	10.60	0.225
P-POSSUM	3	0	-	0.74 (0.67–0.81)	0.15	27.26	0.001
SORT	ps						
SORT v2	3	2	1.5	0.98 (0.97–1.00)	0.004	1.11	0.981

Abbreviations: O: observed; E: expected; AUC: area under curve; 95% CI: 95% confidence interval; H–L: Hosmer–Lemeshow; ps: perfect separation, which is translated into a perfect prediction of mortality; ERCP: endoscopic retrograde cholangiopancreatography; POPF: postoperative pancreatic fistula; Surgical Outcome Risk Tool (SORT); SORT v2; Physiological and Operative Severity Score (POSSUM); Portsmouth-POSSUM (P-POSSUM).

## Data Availability

All original data is available upon request.
